# Optic disc edema associated with neuroborreliosis

**DOI:** 10.1016/j.idcr.2025.e02438

**Published:** 2025-11-29

**Authors:** Molly Barten, Eliisa Strand, Tyler Knight

**Affiliations:** a Indiana University School of Medicine, 635 Barnhill Drive, Indianapolis, IN 46202, United States; b Indiana University School of Medicine, Department of Ophthalmology, 1160 West Michigan Street, Indianapolis, IN 46202, United States

**Keywords:** Lyme Disease, Neuroborreliosis, Optic Disc Edema, Pediatric Ophthalmology

## Abstract

**Purpose:**

Optic disc edema as the primary ocular manifestation of Lyme disease is rare. Current knowledge of the presentation and treatment of optic disc edema in neuroborreliosis relies on case reports. This case presents successful treatment of neuroborreliosis associated optic disc edema in a pediatric patient.

**Observations:**

We present a case of an 11-year-old female who presented to the ophthalmology clinic with a two-month history of headaches and blurry vision. The patient and accompanying guardian reported a preceding history of fever, rash, and presumed cellulitis, which resolved with cephalexin. There was no visual compromise, strabismus or dysmotility, but bilateral mild optic disc edema was seen on dilated fundoscopic exam. A magnetic resonance imaging (MRI) of the brain and orbits was obtained urgently, which revealed optic perineuritis and enhancement of multiple cranial nerves. Bloodwork and cerebrospinal fluid (CSF) studies were later performed, revealing positive serology for Lyme disease. The patient was then prescribed a 14-day course of oral doxycycline. On initial follow up 1-month later, the patient’s subjective blurry vision had resolved, but the optic disc edema had worsened in the left eye. After discussion and shared decision making with the patient’s family, the patient was prescribed a short course of oral prednisone. Follow-up 3 weeks later showed resolution of the optic disc edema and no visual abnormalities, and at delayed follow up there was no recurrence of disc edema and a mild, self-limited headache once or twice weekly was her only symptom.

**Conclusions and Importance:**

This case presents successful treatment of neuroborreliosis associated optic disc edema with doxycycline and oral prednisone in a pediatric patient. Corticosteroids can facilitate the resolution of optic disc edema after antibiotic treatment.

## Introduction

Lyme disease is a bacterial infection caused by the spirochete *Borrelia burgdorferi* and is transmitted to humans through the *Ixodes* tick [1]. Lyme disease is recognized as the most common tick born illness in the United States and Europe [2]. The clinical course of the disease is divided into three stages: first, an early localized presentation with a targetoid rash, erythema migrans, which is pathognomonic for the disease, as well as fever, fatigue, headaches, and arthralgias. Second, an early disseminated disease typically consisting of cranial nerve palsies, meningitis, and carditis. And finally, a late disseminated disease presenting as arthritis [3]. However, the disease can present with any of these manifestations, and erythema migrans is absent 20 % of the time [4]. Ocular manifestations are rare, however they can be seen in all three stages of the disease [5]. The most common ocular manifestations by stage are: first stage – follicular conjunctivitis, second stage—uveitis, optic neuritis, optic atrophy, and/or disc edema, and third stage – stromal/subepithelial keratitis [6].

## Case presentation

An 11-year-old female with past medical history of attention deficit/hyperactivity disorder and anxiety presented to a pediatric ophthalmology clinic in the Midwestern United States with a two-month history of blurry vision and headaches. The headaches were most severe upon awakening in the morning. With targeted questioning, the patient did report pulsatile tinnitus while lying flat. Distance vision had subjectively worsened both with and without her glasses, which were a two-year-old prescription of + 0.75 + 0.50 × 90° OD and + 0.75 SPH OS.

The patient was healthy until three months prior to presentation. At this time, the patient reported a one-week history of fever with right shoulder pain, diffuse myalgias, and malaise and was subsequently seen by her pediatrician. The physical exam was overall unremarkable. Blood work was significant for only an elevated C-reactive protein at 3.7 mg/dL and mildly elevated erythrocyte sedimentation rate (ESR) at 24 mm/hr. Anterior-posterior and lateral X-ray of the humerus and shoulder were without acute pathology. The patient was treated for presumed cellulitis with cephalexin 250 mg every 8 h for 7 days, and the patient’s symptoms resolved. One month later, the patient developed headaches and mild neck stiffness and pain, but these resolved without treatment. An additional month later, the patient developed a painful rash under the left axilla for which the patient received a 7- day course of valacyclovir 1000 mg three times daily presumably to treat atypical herpes zoster, prednisone 40 mg once daily, and cephalexin 500 mg two times daily. The rash resolved shortly after treatment was initiated. Additionally, five days after evaluation at the ophthalmology clinic, the patient reported a heliotrope rash around her left eye and proximal leg pain with weakness using stairs. These signs and symptoms resolved without additional treatment.

The patient was up to date on all vaccinations and the patient’s medication list included amphetamine-dextroamphetamine 7.5 mg twice daily and sertraline 100 mg once daily. Family history was significant for only rheumatoid versus psoriatic arthritis in the patient’s mother. The patient’s social history was significant for living on a rural property with two dogs and a guinea pig. Four months prior to presentation, a tick was found on the patient’s head but was not attached. Additionally, three months prior to presentation, the patient had spent a significant amount of time outside in wooded areas for three weeks in Wisconsin. Although there were no tick bites or ticks noted on the patient, the family’s dog had sixteen ticks removed after this trip.

## Investigations

**Fig. 1:**
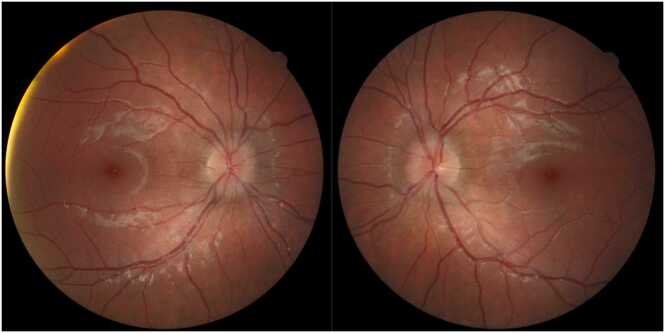
Color fundus photos showing bilateral mild optic disc edema. Right eye (OD) on left screen, Left eye (OS) on right screen – as if patient is looking at you.

**Fig. 2:**
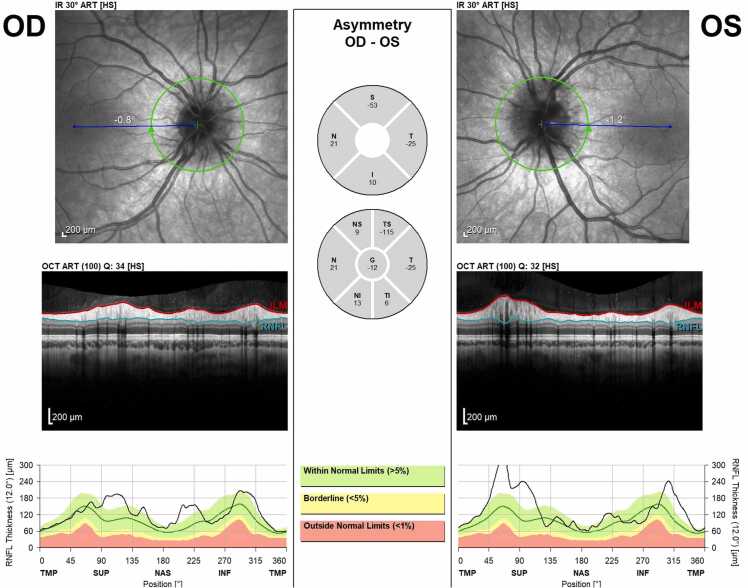
OCT RNFL showing nerve fiber layer edema OS > OD.

**Fig. 3:**
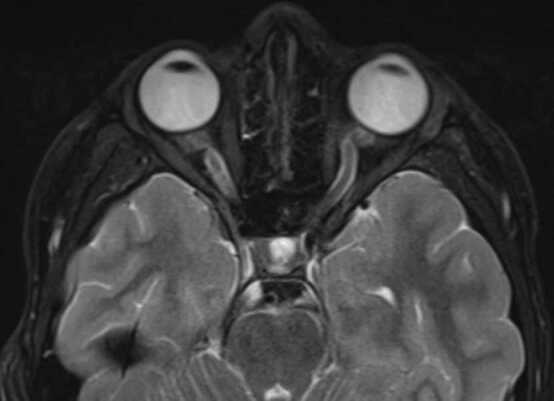
T2 MRI of the brain and orbits showing bilateral optic nerve dilation and enhancement suggestive of optic perineuritis.

The initial complete eye exam was significant for: best corrected Snellen distance visual acuity 20/20 in both eyes, full color plates, no pupil abnormality or afferent pupillary defect, full extraocular movements without strabismus, and a normal anterior slit lamp exam. Fundoscopic imaging showed mild disc edema bilaterally (Fig. 1). The vitreous was clear, the macula was flat, the vessels were of normal course and caliber, and the retina was flat and attached throughout the periphery in both eyes. Optical coherence tomography (OCT) retinal nerve fiber layer (RNFL) analysis revealed more severe edema in the left eye (Fig. 2). An MRI of the brain and orbits with and without contrast demonstrated tortuosity of the infraorbital segment of the left optic nerve and bilateral dilation and enhancement of the optic nerve sheaths suggestive of optic perineuritis (Fig. 3). Additional findings on the MRI included bilateral diffuse enhancement of the oculomotor, trigeminal, abducens, facial, and vestibulocochlear cranial nerves. MRI of the spine demonstrated mild smooth contrast enhancement along the cauda equina nerve roots.

## Differential diagnoses

Prior to the MRI, primary or secondary intracranial hypertension was thought to be most likely, but after the MRI showed enhancement of multiple cranial nerves the differential was broadened significantly. Both inflammatory and infectious processes were considered including bartonella, toxoplasmosis, syphilis, Lyme disease, neurosarcoidosis, and Miller Fisher Syndrome. Neoplastic processes such as leukemia and lymphoma, ischemic processes including anterior ischemic optic neuropathy, and auto immune disorders including rheumatologic diseases (due to the heliotrope like rash and mother’s autoimmune history), neuromyelitis optica (NMO), and myelin oligodendrocyte glycoprotein (MOG) were considered. Enhancement of multiple cranial nerves should include consideration for ingestion of toxins such as methanol and ethylene glycol, chemotherapies, ethambutol, and anti-TNF-alpha medications, but our patient did not have history consistent with any of these diagnoses. Optic neuritis was considered less likely given the patient’s excellent visual acuity. There was also no evidence of any demyelinating lesions on the MRI. The patient’s history of outdoor exposure, fevers, and suspicious rash provided support for testing for Lyme disease and a confirmatory CSF study was pursued.

The following investigations were subsequently performed in the context of our patient’s MRI findings: lumbar puncture with opening pressure and CSF studies: cell count, protein, glucose, culture, meningitis/encephalitis panel, IgG index, clear clonal bands, cytology, flow cytometry, MOG, NMO, arbovirus, Bartonella, Lyme, West Nile, Mycoplasma pneumoniae, and Epstein Barr Virus. Blood work: complete blood count with differential, complete metabolic count, creatine kinase, anti-nuclear antibody, erythrocyte sedimentation rate, angiotensin converting enzyme, Lysozyme, GQ1B (ganglioside) antibodies, and IgG4.

CSF analysis found 59 total nucleated cells (90 % lymphocytes, 10 % plasma cells), < 1 red blood cell, glucose 52 mg/dL, and protein 68 mg/dL. IgM and IgG Lyme antibodies were positive in the CSF. MOG, NMO, Arbovirus, Bartonella, West Nile, Mycoplasma pneumonia, and Epstein Barr virus were negative. Two step Lyme serology testing with ELISA and Western Blot found that 3 of 3 IgM bands were detected and 10 of 10 IgG bands were detected. ESR, ALT/AST, lysozyme, and ANA antibody titer were mildly elevated and are nonspecific findings associated with inflammation due to Lyme disease. CBC and CMP were normal. At this time CRP was normal at 0.6 mg/dL and ESR was elevated at 35 mm/hr. ACE and IgG4 were negative. The patient was referred to both neurology and infectious disease.

## Treatment

The patient was diagnosed with stage 2 early disseminated Lyme disease. After Lyme serology was confirmed, the patient was started on 100 mg doxycycline twice a day for 14 days. After discussion with Infectious Disease, doxycycline was prescribed primarily to prevent progression into a later stage of Lyme disease, understanding it may not necessarily help the optic nerve edema.

## Follow up

**Fig. 4:**
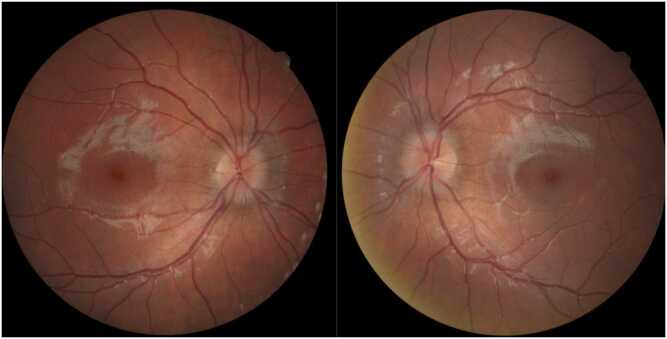
Fundoscopic images after one month from presentation and after a 14-day course of doxycycline. Right eye shows stable/improving disc edema and left eye shows worsening edema.

**Fig. 5:**
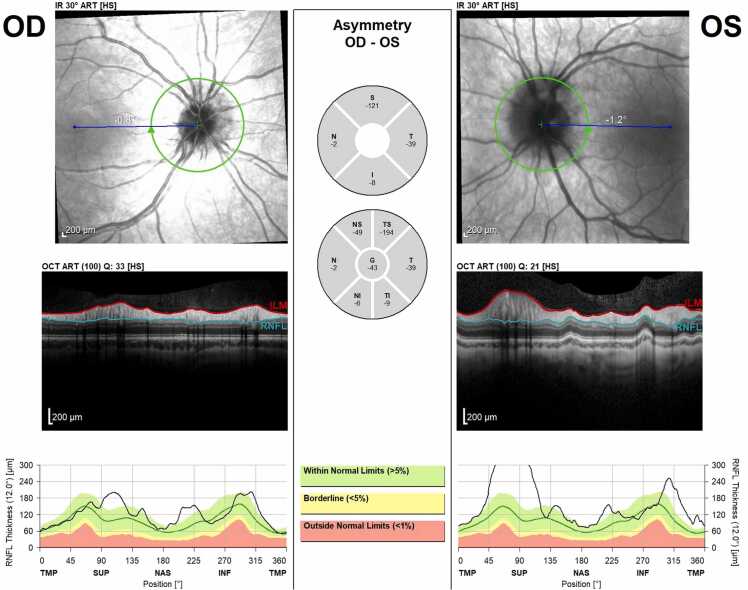
OCT RNFL imaging at one month follow up. Compared to baseline OCT images, edema was overall stable OD, but OS worse with the largest change at the superior aspect of the optic nerve head.

At one month follow up, the patient had completed the 14-day course of doxycycline, and the headaches and blurry vision had resolved. The patient did report buzzing in her ear, intermittent muscle aches in various muscle groups, intermittent radiculopathy/peripheral burning, nerve pain, and diffuse dry skin with peeling, but denied any facial weakness, joint pain, or swelling. Vision was 20/20 in both eyes with full color plates, no afferent pupillary defect, full extraocular movements, and a normal anterior slit lamp exam. The dilated fundus exam was significant for stable optic disc edema in the right eye but worsening optic disc edema in the left eye (Fig. 4). OCT RNFL revealed stable edema in the right eye and worsening edema in the left eye (Fig. 5). After discussion with infectious disease and the patient’s mother, the patient was prescribed 50 mg oral prednisone daily for 2 weeks, followed by a taper decreasing 10 mg weekly until off.

## Outcome and follow up

**Fig. 6:**
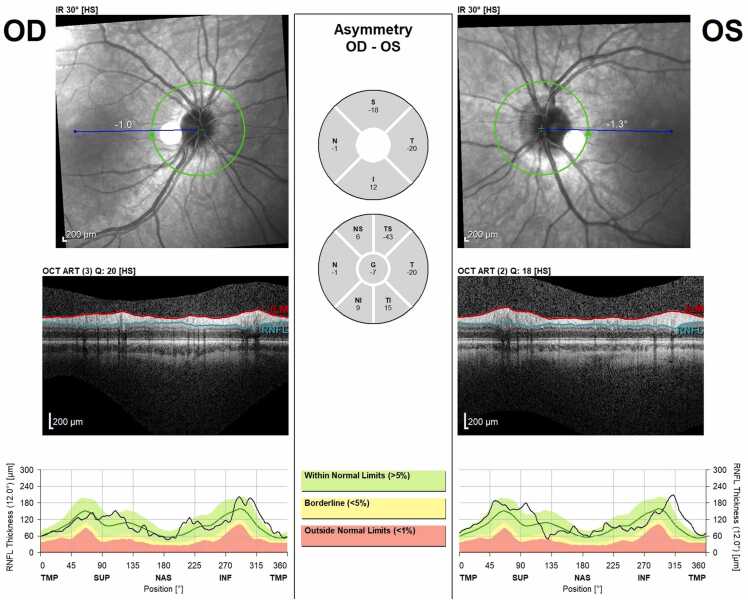
OCT RNFL imaging after 14-day treatment with doxycycline and 18-day treatment with 50 mg prednisone showing interval improvement in both eyes.

At the patient’s next visit 18 days later, the optic disc edema was improving in both eyes on 50 mg oral prednisone daily. The patient tolerated the steroids well without systemic side effects. OCT imaging showed improved edema in both eyes (Fig. 6). The dilated fundus exam showed trace optic disc edema superonasally in the left eye. Prednisone was then tapered by 10 mg every week to off. At extended follow up 6 months from presentation, the patient was medication free, had no optic atrophy, no radiculopathy or joint pain, and had only a mild headache once weekly which resolved without medication.

## Discussion

The diagnosis of Lyme disease is often challenging. Difficulties that clinicians often encounter include the lack of noticing or reporting tick bites and frequent misdiagnoses of erythema migrans, given the varying clinical presentations [7]. Our patient did not recall a tick bite, and her suspicious rash was originally treated as herpes zoster with bacterial superinfection, which makes us suspicious that her rash was likely an atypical presentation of erythema migrans. If earlier stages of the disease are missed, the patient may progress into later stages of the disease which can have devastating long-term consequences.

Neuroborreliosis typically occurs in the second stage of Lyme disease. It is a rare manifestation of the disease and more common in children compared to adults [8]. Neuroborreliosis often presents as facial nerve palsy, meningitis, encephalitis, and/or optic disc edema [9]. Any suspicion for neuroborreliosis should prompt CSF examination for inflammatory changes and antibodies against *Borrelia burgdorferi.* [10] Once the disease is detected through CSF examination, antibiotics should be started immediately. A systematic review of early-stage Lyme neuroborreliosis in adults found that doxycycline and beta-lactam antibiotics such as ceftriaxone were equally effective in treating the disease and equally tolerated [11].

The presentation of neuroborreliosis with optic disc edema is rare and current knowledge relies on case reports. Initial presentation may vary - a systematic review of 11 patients found that 100 % of cases complained of blurry vision, 64 % with headaches, 27 % with scotoma, and 27 % with painful intraocular movements [12]. In a meta-case series of 38 cases of optic disc edema associated with Lyme disease, the mean presenting age was 12.5 years old. Only 27 % of patients had a history of erythema migrans and 27 % recalled a tick bite [13]. In this meta-case series, 23 patients received intravenous ceftriaxone and experienced complete resolution of symptoms, while 8 patients received oral doxycycline. Only 3 patients were treated with corticosteroids, and all three of these patients were concurrently receiving ceftriaxone [14], [15], [16], unlike our patients who received corticosteroids after doxycycline. This meta-case series suggested intravenous ceftriaxone may be associated with better outcomes in the treatment of children with optic disc edema related to neuroborreliosis. However, this often requires hospital admission, PICC line placement, and significant burden to the patient and caregivers. Our case shows a successful outpatient treatment of neuroborreliosis with doxycycline followed by 1 mg/kg oral prednisone followed by taper. The only systematic review investigating outcomes between ceftriaxone and doxycycline included adults with early stage neuroborreliosis. Given the rarity of the condition, additional case reports could help elucidate the ideal treatment options for children with optic disc edema secondary to Lyme disease.

## CRediT authorship contribution statement

**Eliisa Strand:** Writing – review & editing. **Molly Barten:** Writing – review & editing, Writing – original draft, Data curation. **Tyler Knight:** Writing – review & editing, Supervision, Conceptualization.

## Author contribution

Molly Barten: Writing – original draft, chart review and editing. Dr. Eliisa Strand: Writing - review and editing, Dr. Tyler Knight: Writing - review and editing, supervision, conceptualization.

## Consent

Written informed consent was obtained from the patient’s mother for publication of this case report and accompanying images. A copy of the written consent is available for review by the Editor-in-Chief of this journal on request

## Patient consent

Consent to publish this case report has been obtained from the patient’s guardian in writing.

## Ethical approval

Approved.

## Authorship

All authors attest that they meet the current ICMJE criteria for authorship.

## Funding

No funding or grant support.

Research to Prevent Blindness (RPB) provides departmental support for research projects.

## Declaration of Competing Interest

The authors declare that they have no known competing financial interests or personal relationships that could have appeared to influence the work reported in this paper.
